# A comprehensive transfer program from pediatrics to adult care for parents of adolescents with chronic illness (ParTNerSTEPs): study protocol for a randomized controlled trial

**DOI:** 10.1186/s13063-022-06997-0

**Published:** 2022-12-20

**Authors:** Ena Lindhart Thomsen, Kirsten Arntz Boisen, Signe Hanghøj, Helena Hansson, Heidi-Christina V. Grabow Scheelhardt, Susanne Thing Christensen, Bente Appel Esbensen

**Affiliations:** 1 Center of Adolescent Medicine, Department of Paediatrics and Adolescent Medicine, Copenhagen University Hospital – Rigshospitalet, Blegdamsvej 9, 2100 Copenhagen, Denmark; 2 Department of Paediatric and Adolescent Medicine, Copenhagen University Hospital – Rigshospitalet, Blegdamsvej 60B, 2100 Copenhagen, Denmark; 3 Department of Clinical Medicine, Faculty of Health and Medical Sciences, University of Copenhagen, Blegdamsvej 3B, 2200 Copenhagen, Denmark; 4 Copenhagen Center for Arthritis Research (COPECARE), Center of Rheumatology and Spine Disorders, Centre of Head and Orthopaedics, Copenhagen University Hospital – Rigshospitalet, Valdemar Hansens Vej 1, 2600 Glostrup, Denmark

**Keywords:** Adolescent, Chronically ill, Evaluation, Randomized controlled trial, Intervention, Nurse-led support, Nursing, Parents, Protocol, Transitional care

## Abstract

**Background:**

Previous research shows that adolescents with a chronic illness have more successful transfers to adult care if their parents are involved during the transition. However, there is a lack of structured and evaluated transfer programs for parents. Our aim will be to test a comprehensive transfer program for parents of adolescents with chronic illness during the transfer from pediatric to adult care and to evaluate the program’s effectiveness, acceptability, and costs.

**Methods:**

The overall design for this protocol will be a randomized controlled trial. A total of 62 dyads consisting of an adolescent (age 16.5–17.5) and at least one parent will be recruited from one of four pediatric outpatient clinics (nephrology, hepatology, neurology, or rheumatology) at Copenhagen University Hospital - Rigshospitalet, Denmark. The dyads will be randomized to receive the transfer program in addition to usual care or to receive usual care only. The program includes an informative website, bi-annual online educational events, and transfer consultations across pediatric and adult care. Outcome measures will include transition readiness, allocation of responsibility, parental uncertainty level, and transfer satisfaction. Data will be collected from participants at baseline, every 6 months until transfer, at transfer, and 3 months after transfer. The parents’ acceptance of and satisfaction with the program will be explored through semi-structured interviews. Cost, barriers, and facilitators affecting future implementation will be identified in interviews with health care professionals, using the Normalization Process Theory as a framework for the process analysis.

**Discussion:**

To our knowledge, this transfer program is one of the first interventions for parents of adolescents with a chronic illness during their child’s transfer to adult care. Our trial will include parental and adolescent measures allowing us to examine whether a transfer program for parents will improve transfer to adult care for both parents and adolescents. We believe that results from our trial will be helpful in forming recommendations to ensure better involvement of parents in transitional care.

**Trial registration:**

ClinicalTrials.gov NCT04969328. Retrospectively registered on 20 July 2021.

**Supplementary Information:**

The online version contains supplementary material available at 10.1186/s13063-022-06997-0.

## Administrative information

**Table:** 

Title {1}	A comprehensive transfer program from pediatrics to adult care for parents of adolescents with chronic illness (ParTNerSTEPs): Study protocol for a randomized controlled trial.
Trial registration {2a and 2b}.	ClinicalTrials.gov NCT04969328, 20 July 2021, retrospectively registered. https://clinicaltrials.gov/ct2/show/NCT04969328?cond=NCT04969328&draw=2&rank=1
Protocol version {3}	6 December 2022, Protocol version number: 03
Funding {4}	The Novo Nordisk Foundation funds this project in the call “PhD Scholarships in Nursing Research 2019” (grant reference number is NNF19OC0058145).
Author details {5a}	^1^ Department of Paediatrics and Adolescent Medicine, Copenhagen University Hospital - Rigshospitalet, Copenhagen, Denmark. ^2^ Department of Clinical Medicine, Faculty of Health and Medical Sciences, University of Copenhagen, Denmark ^3^Center of Rheumatology and Spine Disorders, Centre of Head and Orthopaedics, Copenhagen University Hospital – Rigshospitalet, Glostrup, Denmark.
Name and contact information for the trial sponsor {5b}	Ena Lindhart Thomsen, Department of Paediatrics and Adolescent Medicine, Copenhagen University Hospital - Rigshospitalet, Blegdamsvej 9, 2100 Copenhagen, Denmark.
Role of sponsor {5c}	The sponsor will be responsible for collecting, managing, analyzing, and interpreting data. The sponsor will also guarantee that the trial complies with the data management plan and ethical guidelines. Novo Nordisk Foundation has no role in the design or evaluation of the trial.

## Introduction

### Background and rationale {6a}

Parents of children with a chronic illness have an important and significant role when it comes to their child’s daily care and treatment. During adolescence, a gradual shift in treatment responsibilities from parents to the adolescent with a chronic illness is expected [1–4].

However, multiple studies have found that parents of adolescents with chronic illness may be reluctant to hand over treatment responsibilities to their child [5–7]. The parents’ difficulties in letting go may prevent a successful transition in which adolescents would be supported in gaining self-determination and medical self-management skills [8, 9].

Lately, there has been an increased focus on transitional care, but studies still find extensive problems with either the clinical setup or patient-related outcomes and experiences:

- The organizational shift from family-centered care in pediatrics to patient-centered care in adult care often impacts families. The differences in focus can make it difficult for parents and adolescents to navigate between the different approaches and expectations [10, 11].
- The transition phase is associated with complications and experiences of exacerbations in the adolescent’s condition [12–14] and an increased number of no-attendance at outpatient clinical visits in adult care [15, 16].
- The transition phase has also been shown to significantly impact parents, including an increased risk of developing anxiety and stress [17–20] and a general concern and sense of uncertainty about their child’s transfer to adult care [21, 22].

Studies have shown that appropriate parental support may increase adolescents’ adherence to treatment [23, 24]. In addition, a close cooperation between parents and health care professionals (HCP) may result in improved health and better transitions for adolescents [21, 25–27]. Parents’ role during the transition phase is also emphasized in transition theories, e.g., the social-ecological model of adolescent and young adult readiness for transition (SMART). Parents’ ability to coach and support their child in gaining medical self-management skills is described as a facilitator of the transition process [8, 28].

In the last decade, the focus of transition programs has been exclusively on adolescents [29, 30], which is why there still is a lack of structured programs to support parents during their child’s transition and transfer to adult health care. Thus, we have developed a comprehensive transfer program for parents of adolescents with chronic illnesses [31]. The program, called ParTNerSTEPs (Parents in Transition – a Nurse-led Support and Transfer Educational Program), aims to support parents during their own transition, in withdrawing and handing over treatment responsibility and to provide them with tools so that they can support their child in the transition phase. Because of the lack of existing parental programs, we have chosen to incorporate the main principles of participatory design to ensure that the parents’ needs are met in the program and that the HCP’s roles are aligned with the clinics’ resources [32]. The transition theory SMART guided the overall focus of the intervention [8]. Finally, the UK Medical Research Council’s (MRC) framework on developing, evaluating, and implementing complex interventions guided the intervention [33]. The framework is useful when dealing with a complex setting, as in our trial, where eight different locations with several interacting components and stakeholders are involved. The MRC framework consists of four key phases: development, piloting, evaluation, and implementation [33]. This protocol will focus only on the evaluation phase, including assessing effectiveness, understanding change process, and assessing cost-effectiveness [33]. A thorough description of the development of ParTNerSTEPs is reported elsewhere [31].

It has been stated that a triple-aim approach should be considered when evaluating transitional care [34]. Triple-aim refers to three interdependent goals: (1) to improve the health of populations, which covers outcomes such as patient-reported outcome measures and self-care skills; (2) to improve the individual experience of health care, including satisfaction with and barriers to care; and (3) to reduce the per capita cost of care, which embraces categories such as the utilization of services and gaps in care.

To our knowledge, ParTNerSTEPs will be one of the first transfer programs for parents. Hence, it is essential not only to estimate the effectiveness but also to examine the delivery of the intervention in a clinical setting and to understand the underlying mechanisms and the program’s acceptability before recommendations can be made, and the implementation phase can start.

### Objectives {7}

The overall aim of this randomized controlled trial (RCT) is to test and evaluate ParTNerSTEPs (Parents in Transition – a Nurse-led Support and Transfer Educational Program).

- To evaluate the ParTNerSTEPs program’s effectiveness on adolescents’ transition readiness (TR), as assessed by the parents and adolescents themselves
- To explore the acceptability of and satisfaction with the ParTNerSTEPs program (including facilitators and barriers) among parents in the intervention group
- To identify costs, barriers, and facilitators affecting the future implementation of ParTNerSTEPs in practice

### Hypotheses

We believe that we will be able to improve the transition process for adolescents with a chronic illness by preparing and supporting their parents. Thus, we hypothesize that, compared to the control group, the participants in the intervention group will report significantly higher transition readiness scores, a higher level of adolescent responsibility, lower parental uncertainty scores, and higher transfer satisfaction.

### Trial design {8}

The trial design is an RCT with parallel groups of one intervention arm (ParTNerSTEPs + usual care) compared with a control arm (usual care) (1:1). This protocol has been drafted in accordance with the Standard Protocol Items: Recommendations for Interventional Trials (SPIRIT). The SPIRIT 2013 checklists can be found in Additional file 1.

## Methods: participants, interventions, and outcomes

### Study setting {9}

The intervention will be carried out at the nephrology, hepatology, neurology, and rheumatology pediatric and adult outpatient clinics at Copenhagen University Hospital - Rigshospitalet, Denmark. In Denmark, young people’s transfer to adult care takes place when they turn 18 years old, regardless of their maturity or transitional readiness.

### Eligibility criteria {10}

Participants are parents, stepparents, or guardians (subsequently referred to as parents) of adolescents affiliated with one of the four abovementioned pediatric outpatient clinics. Parents who meet the following criteria will be invited to participate in the trial.

*Inclusion criteria:* parents of adolescents with chronic illness who (1) are aged 16.5–17.5; (2) have been diagnosed for minimum 6 months; (3) have regular check-ups at the Department of Paediatrics and Adolescent Medicine’s nephrology, hepatology, neurology, or rheumatology outpatient clinics at Copenhagen University Hospital - Rigshospitalet, Denmark; (4) will be transferred to an adult hospital department at Rigshospitalet; and (5) are cognitively able to take responsibility for their treatment.

*Exclusion criteria*: (1) parents who do not read and speak Danish and (2) parents of adolescents who will be transferred to their family doctor upon reaching the age of 18.

### Who will take informed consent? {26a}

Parents will be asked to give informed consent to participate in the trial. Adolescents will not be asked to sign a written informed consent as they are under 18. The project coordinator will provide oral and written information about the trial before informed consent is obtained.

### Additional consent provisions for collection and use of participant data and biological specimens {26b}

On the consent form, participants will be asked if they will allow us to contact them regarding a follow-up interview. This trial does not involve collecting biological specimens for storage.

## Interventions

### Explanation for the choice of comparators {6b}

We aimed to test ParTNerSTEPs in a clinical setting and to examine whether a transfer program for parents together with the usual care for the adolescent will result in a more successful transfer. For that reason, we will choose families from the same outpatient clinics as comparators. Participants in the control group will only receive the usual transitional care targeting adolescents, in which parents are not systematically involved.

### Intervention description {11a}

**Fig. 1:**
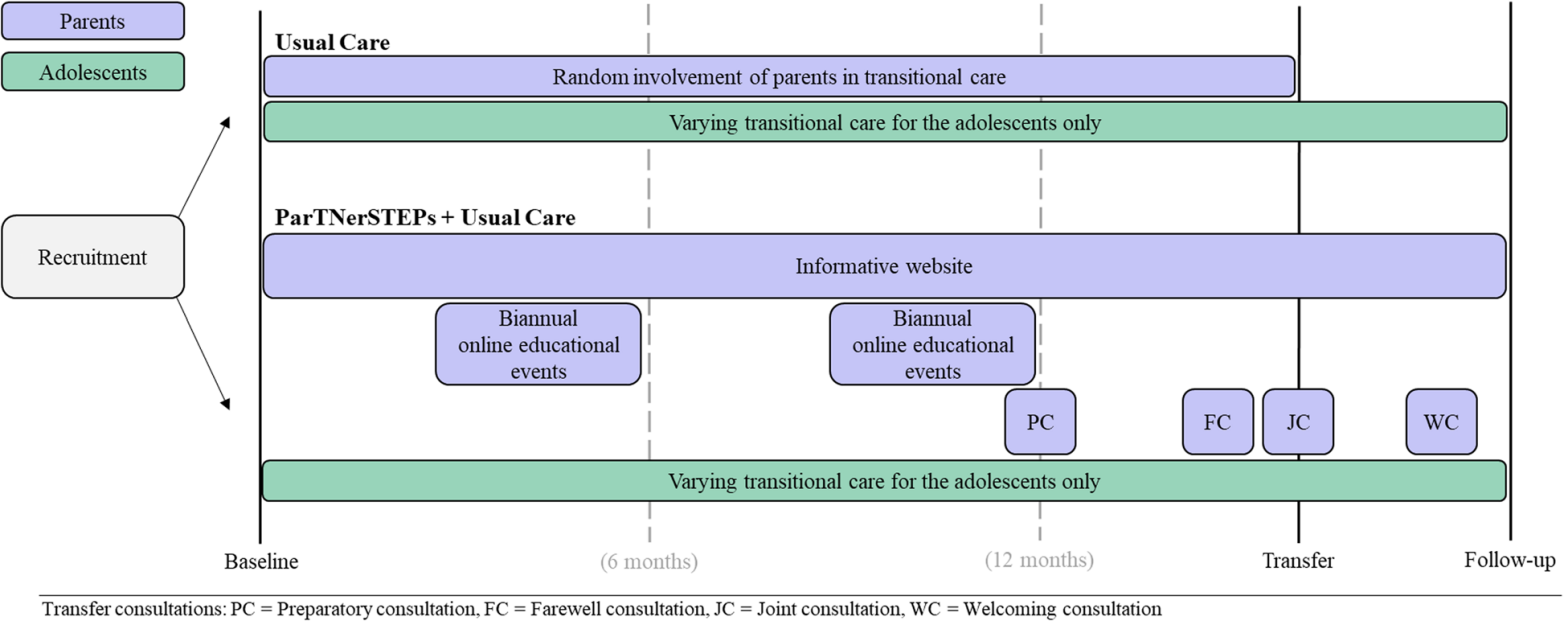
Model of the ParTNerSTEPs program intervention

The intervention group will receive the ParTNerSTEPs program, described in detail elsewhere [31]. ParTNerSTEPs consists of three components: (1) an informative website, (2) online educational events for parents, and (3) transfer consultations with providers from both pediatrics and adult care (Fig. 1).

The ParTNerSTEPs program will be offered to the parents from 6 to 18 months before their child is transferred to adult care (depending on the adolescent’s age at recruitment). *The website* contains, among other things, information about legal changes, advice, and guidance on how parents can prepare their child and an introduction to the adult departments. The website will be available for parents immediately after recruitment and can be used freely throughout the intervention [31]. *The online educational events* will be offered twice a year as a webinar. There will be short presentations on different topics, such as young people’s experiences with the transfer and the possibility of talking to nurses from the adult care team in disease-specific breakout rooms [31]. *The transfer consultations* across the pediatric and adult departments consist of four individual consultations: (1) a preparatory split-visit consultation with the pediatric nurse, which will take place 3–6 months before transfer; (2) a farewell consultation with the pediatric nurse and/or doctor at the last consultation in pediatrics; (3) a joint consultation at transfer, where both the pediatrician and adult physician will be present; and (4) a welcoming consultation with the adult care nurse 0–3 months after transfer. Both the parents and the adolescent will be present during the consultations [31].

### Criteria for discontinuing or modifying allocated interventions {11b}

ParTNerSTEPs will be offered to all participants in the intervention group, but the families are free to choose whether they will use all or just some of the elements. The intervention will be discontinued if the participant revokes their consent to participate in the trial or if the adolescent moves to another region, as it affects the transfer to the included adult clinics.

### Strategies to improve adherence to interventions {11c}

All HCP associated with the project will receive training in the intervention before implementation. To improve adherence, HCP who conducts the consultations will receive a manual with detailed information about the four consultations, in which the aim, content, procedure, and subsequent documentation are described. The manual can be forwarded by contacting the first author. All nurses have or will receive training in developmentally appropriate care and communication with adolescents and will receive supervision during and after their first consultation.

### Relevant concomitant care permitted during the trial {11d}

In all four sites, the adolescent will receive transitional care of varying degrees. Transitional care can include split-visit consultations [35], where HCP spend time alone with the adolescent and with the adolescent and parents. None of the sites will offer additional support to the parents. However, we are aware that parents allocated to the control group may be disappointed at not being offered support. In such cases, we will inform the parents that their child will still receive standard transitional care in the clinic. We will also encourage them to contact HCP at their respective outpatient clinic to discuss the transfer.

### Provisions for post-trial care {30}

Not applicable—as there is no anticipated harm and compensation for trial participation.

### Outcome {12}

The evaluation process will be multi-method (questionnaires and interviews), with multi-informants (parents, adolescents, and HCP) to answer the various objectives in our trial.

Quantitative data will be collected through questionnaires at baseline, at transfer, and at 3 months after the adolescent’s transfer to adult care (follow-up). Depending on the adolescent’s age at recruitment, data will also be collected every 6 months until transfer (Table 1). The time of transfer will be registered the day after the adolescent’s last consultation in the pediatric department.

**Table 1:** Overview of the timeframe for data collection

Outcomes and questionnaires	Time frame				
	Baseline	6 months	12 months	Transfer	3-month follow-up
**Socio-demographic**					
Parents	X				
**Transition readiness**					
Parents	X	(X)	(X)	X	X
Adolescent	X	(X)	(X)	X	X
**Allocation of responsibility**					
Parents	X	(X)	(X)	X	X
Adolescent	X	(X)	(X)	X	X
**Uncertainty**					
Parents	X			X	
**Health-related quality of life**					
Adolescent	X			X	X
**Experiences of transfer**					
Parents					X
**Transfer satisfaction**					
Parents					X
Adolescent					X

The measures that will be used to estimate the ParTNerSTEPs program’s effectiveness are presented below.

#### Primary outcome

##### Transition readiness (TR)

TR will be measured by the questionnaire Medical self-management and transition readiness, developed and validated by Williams et al. [36]. The TR questionnaire will be completed by parents (primary outcome) and adolescents (secondary outcome).

#### Main secondary outcome

##### Allocation of responsibility (AoR)

AoR will be measured by the Allocation of Responsibility questionnaire developed and validated by Bilhartz et al. [37]. Parents and adolescents will complete the questionnaire.

##### Uncertainty

The Uncertainty Scale will measure parental uncertainty. The scale was developed and validated by Burström et al. [38]. The Uncertainty Scale will be completed only by the parents.

##### Health-related quality of life 

Health-related quality of life will be measured by EuroQual-5-Domain (EQ-5D-5L) [39]. EQ-5D-5L will be completed only by adolescents.

##### Experience of transfer 

Transfer experiences will be measured by a questionnaire that has been self-developed by the research group. The questionnaire will be completed only by parents.

##### Transfer satisfaction

A self-developed satisfaction scale will measure transfer satisfaction. Both parents and adolescents will complete the scale.

### Participant timeline {13}

**Fig. 2:**
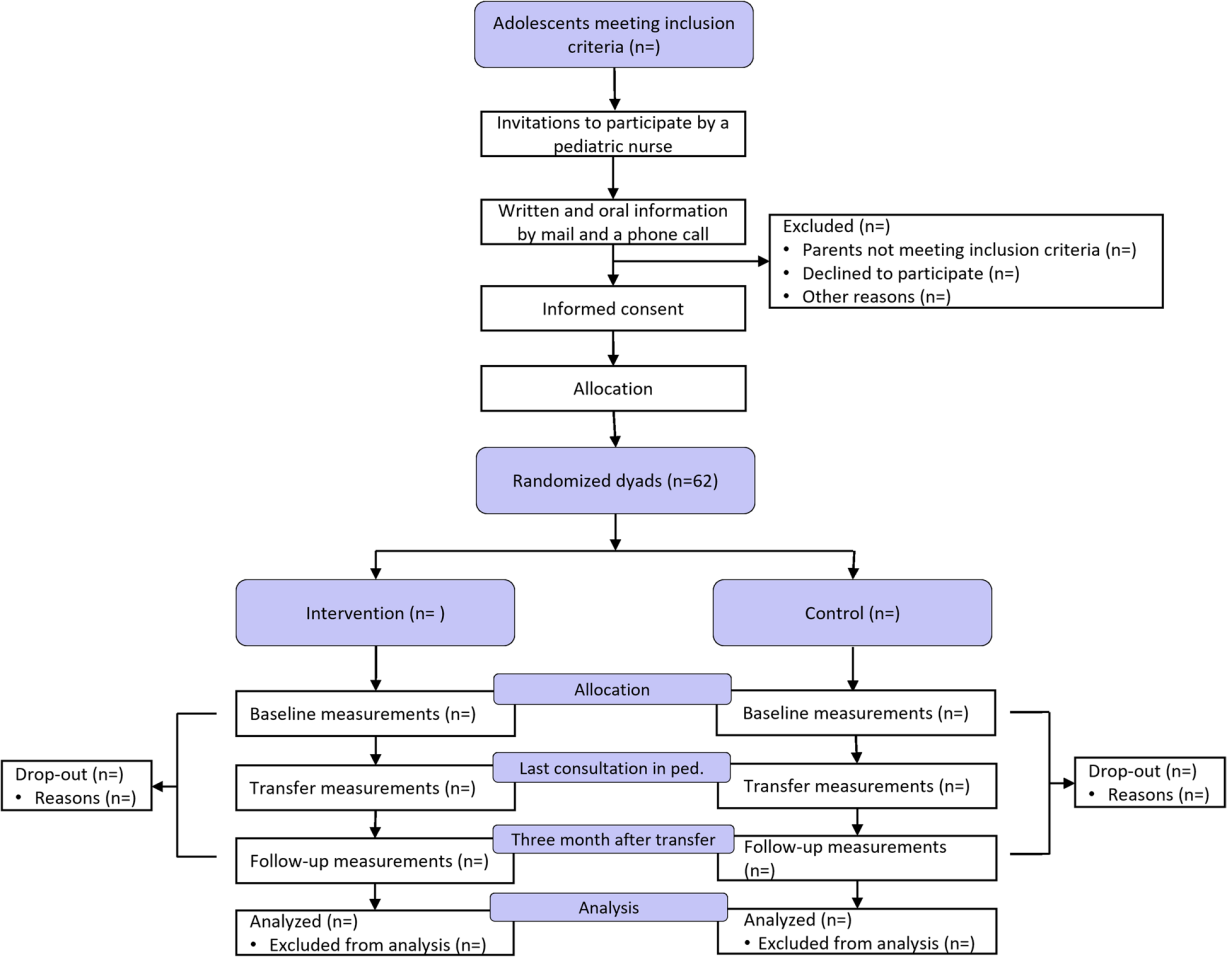
Flow of participants

The participants will be allocated to either the control or the intervention group immediately after enrollment and signing informed consent. They will be asked to complete questionnaires at baseline, at transfer, and at 3 months after the adolescent’s transfer to adult care (follow-up), regardless of which group the participant is allocated to. A flowchart of the participants’ timeline is shown in Fig. 2.

Participants in the intervention group will additionally be invited to biannually online educational events and four transfer consultations. See Fig. 1 for more information.

### Sample size {14}

No previous studies have focused on whether it is possible to increase adolescents’ TR, as assessed by the parents, by offering the parents a comprehensive transfer program. A previous cross-sectional study found that parents of chronically ill adolescents have an average TR score of 3.12 (standard deviation (SD) 0.68) [36]. Based on our clinical experience, corresponding with the author of the primary outcome questionnaire by email, and a review of each item in the questionnaire (Medical self-management and transition readiness) [36], we expect that ParTNerSTEPs will be able to increase the parents’ TR score in the intervention group by 0.5 points.

We are planning an RCT with a continuous response variable from independent control and intervention groups with one control per intervention subject. Based on the abovementioned cross-sectional study, we assume the response within each group will be normally distributed with standard deviation 0.68. If the true mean difference between the intervention and control groups is 0.50, we will need to include 31 dyads of one adolescent and at least one parent in each group to be able to reject the null hypothesis that the population means of the intervention and control groups are equal, with probability (power) 0.80. The type I error probability associated with the test of this null hypothesis is 0.05. Thus, we need to recruit 62 dyads (31 in each group).

### Recruitment {15}

Potential participants will be contacted by pediatric nurses at the four outpatient clinics and briefly informed about the trial. The project coordinator will provide parents interested in participation with oral and written information about the trial, including the fact that they can withdraw from the trial at any time without consequences for their child’s treatment. The adolescent will also be provided with written information and be offered the opportunity to speak directly to the project coordinator if they have any questions. We will carefully consider the risk of coercion, autonomy, and confidentiality during the recruitment process. The recruitment period will start in July 2021 and is expected to end primo 2023.

## Assignment of interventions: allocation

### Sequence generation {16a}

When recruited, the participants will be divided into family dyads of an adolescent and at least one of the parents. The family dyads will be randomized into two groups: (1) the intervention group (usual care plus ParTNerSTEPs until the adolescent is transferred to adult care) or (2) the control group (usual care only). Randomization will be computer-generated and conducted by a random number algorithm in REDCap (Research Electronic Data Capture) with a ratio of 1:1 (stratified by the four outpatient clinics and with block sizes of 4–6). A person not involved in the recruitment or the trial will create the algorithm and block sizes. The block sizes and algorithm will be blinded for all HCP and the project coordinator. Once the database has defined a dyad’s allocation, no changes can be made.

### Concealment mechanism {16b}

The allocation list will be concealed in REDCap, where only the project coordinator can access it. HCP conducting the transfer consultations will be informed of participants allocated to the intervention. Participants allocated to the control group will be concealed from all HCP.

### Implementation {16c}

The project coordinator will enroll the participants over the phone and enter the participants’ information in REDCap. When all data is collected, REDCap will generate the allocation of the family dyad to either the intervention or control group. The family will immediately be informed of their allocation.

## Assignment of interventions: blinding

### Who will be blinded {17a}

Due to the nature of this trial, it is impossible to blind the participants or HCP. Thus, the trial is an open-label trial, and no blinding will occur. Data analysts will be blinded.

### Procedure for unblinding if needed {17b}

The trial is open-label and consequently unblinding will not occur.

## Data collection and management

### Plans for assessment and collection of outcomes {18a}

All quantitative data will be self-reported by the participants, which is why no assessors are needed in this trial. The validity of the data will be secured by only using validated questionnaires, which are translated and cross-cultural adapted after guidelines proposed by Wild [40].

The primary outcome TR will be measured by the questionnaire Medical self-management and transition readiness [36]. It consists of 21 items addressing adolescents’ awareness of their health condition and ability to make decisions relevant to their health care needs. Participants will be asked to respond to each item on a 5-item Likert scale (1=strongly disagree, 2=disagree, 3=neither disagree nor agree, 4=agree, or 5=strongly agree). A high overall score indicates higher transition readiness.

Secondary outcomes include (i) AoR, which will be measured by the questionnaire Allocation of Responsibility [37]. AoR consists of 13 items. Participants can choose from one of four response statements: (1) parent/guardian takes primary responsibility, (2) responsibility is shared between the parent/guardian and the adolescent, (3) adolescent takes primary responsibility, or (0) not applicable or no one does this. A high total score indicates a high level of adolescent responsibility. (ii) Uncertainty will be measured by the Uncertainty Scale [38]. The scale measures parental uncertainty during their child’s transfer from pediatric to adult care on a linear analog scale from 0 (not uncertain at all) to 100 (extremely uncertain). A high score indicates a high level of uncertainty. (iii) Health-related quality of life will be measured by EQ-5D-5L [39]. The questionnaire comprises five dimensions: mobility, self-care, usual activities, pain/discomfort, and anxiety/depression. Each dimension has five levels: no problems, slight problems, moderate problems, severe problems, and extreme problems. A low total score indicates better health-related quality of life. (iv) Experience of transfer will be measured by a self-developed questionnaire. The questionnaire has been content and face validated by parents in the development phase [31]. The questionnaire consists of 11 items, based on the modifiable factors in the SMART transition theory [8]. Participants will be asked to respond on a 5-item Likert scale (1=strongly disagree, 2=disagree, 3=partially agree, 4=agree, and 5=strongly agree) (Additional file 2). A high overall score indicates a positive experience of transfer. (v) Transfer satisfaction will be measured by a single question: “On a scale of 0-100, how satisfied have you been with your/your child's transfer to the adult department?” The scale is a linear analog scale from 0 (very unsatisfied) to 100 (very satisfied). A high score indicates a high level of satisfaction. All the questionnaires will take approximately 10 min to complete.

Process evaluation is a suitable method to explore the acceptability and satisfaction of the intervention (objective 2) and thereby gain a deeper understanding of what makes an intervention effectful, what the challenges are, and how it can be optimized [33, 41]. Both quantitative and qualitative data will be used to examine the parents’ acceptance of and satisfaction with the ParTNerSTEPs program. Quantitative data will be collected at the 3-month follow-up, with questions regarding the use of the program, including how often the website was visited and possible reasons for not attending online educational events or transfer consultations (see Additional file 3). The parents’ perceptions of the program (including facilitators and barriers for using the program) will be explored in semi-structured interviews with parents from the intervention group (*n*=10–12). The final development of the interview guide will be based on results from the quantitative data.

Assessing the costs in relation to the intervention is a crucial step before the implementation phase can start [41]. To identify costs, barriers, and facilitators affecting future implementation (objective 3), we will conduct structured interviews with HCP from pediatric and adult care. The interviews will focus on the HCP’s perceptions of the program, their role in the program, and their working hours in the project (including preparation and medical record-keeping). We aim to interview physicians, nurses, and management from all four sites (*n*=16).

### Plans to promote participant retention and complete follow-up {18b}

An online link to the questionnaires will be sent out via email. Two automatic email reminders will be sent in the following 2 weeks. If the participants have not answered the questionnaires, a text message will be sent with the link, and/or a phone call will be made to the participant.

### Data management {19}

The quantitative data will be collected through a private link sent by REDCap, a double password-protected software. Thus, all data will be entered by the participants themselves and pseudonymized before being transferred to an analysis program (R version 4.1.2). The qualitative interviews will be audio-recorded and subsequently transcribed verbatim. Personally identifiable data will be anonymized in accordance with Danish law. All audio and transcribed data will be processed in accordance with the data protection plan. The Danish Data Protection Agency approves the plan, reference no. VD-2018-396.

### Confidentiality {27}

All data will be handled confidentially and stored in the password-protected software REDCap without connection to the adolescents’ medical journal. Only the project coordinator can access REDCap. Participants will be allocated to an individual family dyad trial identification number immediately after randomization.

### Plans for collection, laboratory evaluation, and storage of biological specimens for genetic or molecular analysis in this trial/future use {33}

Not applicable. No biological specimens will be collected (see 26b).

## Statistical methods

### Statistical methods for primary and secondary outcomes {20a}

Quantitative data will be analyzed using the statistical computing software R version 4.1.2. A detailed statistical analysis plan will be developed in collaboration with a statistician before all participants have completed the final measurement. The primary aim is to compare the intervention and the control groups. We plan to use *t*-tests, linear regression, and linear mixed models with both fixed and random effects, as appropriate. Model assumptions will be checked. Regression analyses will be adjusted for the following variables: age and gender of the adolescent, the parent’s gender, and baseline scores. The primary outcome will be the difference in TR, as assessed by the parents, from baseline to transfer. Separately, a similar analysis will be conducted using the difference in TR assessed by the adolescents. Likewise, analysis with AoR, uncertainty, and health-related quality of life will be conducted. The statistical analysis plan will be confirmed among all project group members before we examine the data. Data will be presented as percentages, means with 95% confidence intervals, *p*-values, and graphics.

Data regarding the parents’ experiences and satisfaction with the transfer will be analyzed using descriptive statistics, including the frequency distribution and central tendency.

### Interim analyses {21b}

Not applicable, as there are no anticipated problems that are detrimental to the participants.

### Methods for additional analyses (e.g., subgroup analyses) {20b}

Qualitative data will be handled and analyzed using the qualitative data software QSR NVivo v20.6.1.1137. All interviews will be audio-recorded and subsequently transcribed verbatim. Data from objective 2 will be analyzed using a thematic analysis approach inspired by Braun and Clarke’s 6-step model [42]. Data regarding the transfer program’s feasibility (objective 3) will be analyzed using the Normalization Process Theory framework [43].

### Methods in analysis to handle protocol non-adherence and any statistical methods to handle missing data {20c}

Our primary analysis population will be all parents and adolescents with available data at baseline, transfer, and 3-month follow-up. We will attempt to follow up all randomized parents and adolescents and therefore expect minimal loss to follow-up and missing data.

### Plans to give access to the full protocol, participant-level data, and statistical code {31c}

The datasets generated and analyzed during the current trial are not publicly available but are available upon request to the first author (ET).

## Oversight and monitoring

### Composition of the coordination centre and trial steering committee {5d}

During the development of the trial, a steering group consisting of researchers, clinicians, and patient representatives was set up. The appointed HCP from each site will be responsible for implementing the intervention with support from the project coordinator. All HCP involved in the trial will report obstacles to the project coordinator as soon as possible. Rigshospitalet’s Department of Paediatrics and Adolescent Medicine will, together with the steering group, provide a team for the objective evaluation of the intervention.

### Composition of the data monitoring committee, its role, and reporting structure {21a}

Not applicable, as the trial is estimated to be a low-risk intervention.

### Adverse event reporting and harms { 22}

We do not expect parents or adolescents to be harmed by participating in the intervention.

### Frequency and plans for auditing trial conduct {23}

Not applicable—as a thorough process evaluation will take place parallel to the conduction of the trial.

### Plans for communicating important protocol amendments {25}

Any deviations from the protocol will be fully documented using a breach report form. We will report protocol modifications and relevant process changes in the trial registration site and notify the sponsor.

### Dissemination plans {31a}

Trial results will be disseminated through scientific publications, professional conferences, and written reports. All involved HCP and participants, who have stated in their informed consent that they want to be informed, will receive a summary of the results from the trial.

## Discussion

In recent years, the focus on transition and transfer programs for adolescents with a chronic illness has expanded in both the clinical setting and the field of research [29, 30, 44]. Although parents play a significant role in treating and caring for adolescents with a chronic illness, there is still a lack of structured transfer programs for parents. To our knowledge, ParTNerSTEPs is the first parent-focused transfer intervention designed as an RCT. Our intervention is based on the hypothesis that a nurse-led transfer intervention targeting parents will improve parents’ readiness for their child’s transfer to adult health care and strengthen the adolescent’s self-management skills. Our setup, in which data will be collected from both the parents and the adolescents, will allow us to examine whether ParTNerSTEPs is effective in increasing adolescents’ TR, as assessed by the parents and adolescents themselves; reducing parental uncertainty; and facilitating a shift in treatment responsibility from parents to the adolescent.

The transition process is a complex issue, as it takes place in outpatient clinics across pediatric and adult care and involves multiple elements and stakeholders, such as adolescents, parents, and HCP with different backgrounds. Based on this, we have chosen MRC’s framework for developing and evaluating a complex intervention [33]. During the development phase, we also used participatory design to involve relevant stakeholders in the development of the program [32, 45]. Our close collaboration with the stakeholders during the development phase gave us a unique insight into the users’ needs and experiences, which is why we also plan to involve the collaboration group in the evaluation phase, e.g., by inviting them to contribute with topics for the interview guides, etc.

During the planning of the evaluation phase, a new MRC framework for developing and evaluating complex interventions was published [46]. Even though a newer framework was available, we decided to adhere to the MRC framework from 2013 for the following reasons: (1) the ParTNerSTEPs program was developed based on the framework from 2013, (2) the first participants were included and randomized before the publication of the new framework, and (3) we believe that the transparency for the entire trial would be lessened if we chose to change the overall framework mid-way. However, we have studied the new framework closely and believe that we have followed the development, intervention, feasibility, and evaluation phases and incorporated the core elements in the trial [31].

### Limitations

A part of the intervention (the transfer consultations) will occur in the clinic, thus relying on HCP’s participation and willingness to adhere to the consultation scripts. Therefore, daily clinical issues, such as staff shortages, problems in interprofessional cooperation, and time pressure, might hinder the HCP in conducting the intervention as described. We have tried to prevent this by including the HCP in the development phase, where discussions of realistic workflows and resources were addressed. We will also be in close contact with the HCP during the intervention to detect and act on possible barriers/challenges.

Despite providing adequate scripts for the consultations in ParTNerSTEPs and training the involved HCP in the intervention, the complexity of our intervention (including multiple components, multiple stakeholders, and different settings) might considerably affect our results. For example, the degree of motivation for conducting the consultations can vary among HCP, which may impact how the family benefits from the consultations. We will address this area in our discussion of the major findings.

## Conclusions and impact

In conclusion, this trial protocol describes the design of an RCT that aims to test a comprehensive transfer program in a clinical setting and to evaluate the program’s effectiveness, acceptability, and feasibility. The trial is clinically important because it aims to generate high-quality evidence for how parents can be involved and supported during their child’s transfer to adult care. We expect that our trial results may inform future recommendations to ensure better involvement of parents in the transition process.

### Trial status

This is protocol version 3, 6 December 2022. ClinicalTrials.gov ID NCT04969328. First registered on June 30, 2021; met QC criteria on July 9, 2021; last update posted on July 20, 2021. Recruitment started on July 14, 2021, with an anticipated primary completion date of January 2023.

## Supplementary Information

**Additional file 1.** SPIRIT 2013 Checklist.

**Additional file 2.** Experiences of the transfer.

**Additional file 3.** Evaluation of the ParTNerSTEPs program.

**Additional file 4.** Consent form for participating in ParTNerSTEPs.

## Data Availability

The dataset will be available upon reasonable request once the results have been published.

## Abbreviations

HCP: Health care professionals
SMART: Social-ecological Model of Adolescent and young adult Readiness for Transition
ParTNerSTEPs: Parents in Transition – a Nurse-led Support and Transfer Educational Program
MRC: Medical Research Council
RCT: Randomized controlled trial
TR: Transition readiness
SPIRIT: Standard Protocol Items: Recommendations for Interventional Trials
AoR: Allocation of responsibility
EQ-5D-5L: EuroQual-5-Domain
REDCap: Research Electronic Data Capture
