# Supplementary material for: A comprehensive transfer program from pediatrics to adult care for parents of adolescents with chronic illness (ParTNerSTEPs): study protocol for a randomized controlled trial
Source: Trials. 2022 Dec 20;23:1034. doi: 10.1186/s13063-022-06997-0 (PMC9768961; doi:10.1186/s13063-022-06997-0)
Supplement: Supplementary file 2 — Additional file 2. Experiences of the transfer. [file 13063_2022_6997_MOESM2_ESM.doc]

**Additional file 2**

# Your experiences of the transfer

Please use the following scale to answer the statements below.

Mark how much you agree with each statement.

|  | Strongly  disagree | Disagree | Partially  agree | Agree | Strongly  agree |
| --- | --- | --- | --- | --- | --- |
| I experience that my child and I have been adequately prepared for the transfer to the adult department | **1** | **2** | **3** | **4** | **5** |
| I feel my child was ready to leave pediatrics and transfer to the adult department | **1** | **2** | **3** | **4** | **5** |
| I know the benefits of my child’s transfer to the adult department | **1** | **2** | **3** | **4** | **5** |
| I have come to terms with my child’s transfer to the adult department | **1** | **2** | **3** | **4** | **5** |
| I found that my child and I were involved in planning the best possible transfer of my child | **1** | **2** | **3** | **4** | **5** |
| I found that the staff from pediatric and adult care collaborated around my child's transfer | **1** | **2** | **3** | **4** | **5** |
| I trust that the adult team has sufficient knowledge of my child's medical history | **1** | **2** | **3** | **4** | **5** |
| I am confident that the adult department can manage the treatment of my child's illness | **1** | **2** | **3** | **4** | **5** |
| I support my child in managing the illness him/herself | **1** | **2** | **3** | **4** | **5** |
| I have sufficient knowledge to support my child in educational issues | **1** | **2** | **3** | **4** | **5** |
| I have sufficient knowledge of social services legislation for young people over the age of 18 | **1** | **2** | **3** | **4** | **5** |
