# Supplementary material for: A comprehensive transfer program from pediatrics to adult care for parents of adolescents with chronic illness (ParTNerSTEPs): study protocol for a randomized controlled trial
Source: Trials. 2022 Dec 20;23:1034. doi: 10.1186/s13063-022-06997-0 (PMC9768961; doi:10.1186/s13063-022-06997-0)
Supplement: Supplementary file 3 — Additional file 3. Evaluation of the ParTNerSTEPs program. [file 13063_2022_6997_MOESM3_ESM.doc]

**Additional file 3**

# Evaluation of the ParTNerSTEPs program

**WEBSITE**

How often have you used the website 'ParTNerSTEPs'?

- Never
- Once or twice
- Around 3-10 times
- 11-20 times
- More than 20 times

Which pages were relevant to you? (You can choose several options)

- What is good to know before my child turns 18?
- How can I prepare myself and my child during the transition?
- What can I and my child expect of the adult department?
- What do other parents and adolescents say about the transition?
- Questions and answers
- None of the pages were relevant to me

**ONLINE EDUCATIONAL EVENTS**

Have you taken part in one or more educational events?

- No
- Yes

If yes, how many educational events have you taken part in? ______________________________________

If no, what was the primary reason for not participating?

- Had a hard time prioritizing it
- The agenda was not relevant to me
- Did not feel like participating
- The time did not suit me
- Other

Please explain why you did not participate_______________________________________________________________

**TRANSFER CONSULTATIONS**

How relevant were the various consultations for you and your child on a scale of 1-5?

|  | I did not participate in the consultation | Not relevant at all | Not relevant | A little relevant | Relevant | Very relevant |
| --- | --- | --- | --- | --- | --- | --- |
| **The preparatory consultation** | 0 | 1 | 2 | 3 | 4 | 5 |
| **The farewell consultation** | 0 | 1 | 2 | 3 | 4 | 5 |
| **The joint consultation** | 0 | 1 | 2 | 3 | 4 | 5 |
| **The welcoming consultation** | 0 | 1 | 2 | 3 | 4 | 5 |

What is the reason for not participating in one or more of the consultations?

- My child's other parent participated instead of me
- I was prevented from participating (e.g., due to illness, work or other)
- I was not offered the consultation
- I did not find it relevant to participate
- Other

If other, please explain why you did not participate _____________________________________________________
