# Supplementary material for: A comprehensive transfer program from pediatrics to adult care for parents of adolescents with chronic illness (ParTNerSTEPs): study protocol for a randomized controlled trial
Source: Trials. 2022 Dec 20;23:1034. doi: 10.1186/s13063-022-06997-0 (PMC9768961; doi:10.1186/s13063-022-06997-0)
Supplement: Supplementary file 4 — Additional file 4. Consent form for participating in ParTNerSTEPs. [file 13063_2022_6997_MOESM4_ESM.doc]

**Consent form for participating in ParTNerSTEPs**

**Informed consent to participate in a health science research project:**

**ParTNerSTEPs: Parents in Transition – a Nurse-led Support and Transfer Educational Program**

**Statement from participant:**

I have received written and oral information, and I know enough about the project to be able to say yes to participating.

I am informed that participation is voluntary, and I can withdraw my consent at any time without it affecting my child's treatment.

I give consent to participate in the research project and have received a copy of this consent sheet as well as a copy of the written information about the project for my own use.

Participant’s full name:­­_____________________________ Social Security No.:_______________

My child’s full name: _______________________________Social Security No.:_______________

Date:______________ Signature:_____________________________________________________

Do you wish to be informed about the results of the research project?

Yes _______ No ______

May we contact you later regarding a possible follow-up interview?

Yes _______ No ______

**Statement from project manager:**

I confirm that the participant has received oral and written information and that the participant has had the opportunity to ask questions about the project.

In my opinion, sufficient information has been provided to enable a decision to be taken on participation in the project.

Project manager’s name: ___________________________________________________________

Date:______________ Signature:_____________________________________________________
